# Tibio‐tarsal arthrodesis in Italy: Results from an epidemiological 16‐year nationwide study

**DOI:** 10.1002/jeo2.70302

**Published:** 2025-06-15

**Authors:** Umile Giuseppe Longo, Rocco Papalia, Alessandro Mazzola, Alessandra Corradini, Alessandro De Sire, Pieter D'Hooghe, Ilaria Piergentili, Kristian Samuelsson, Stefano Zaffagnini, Vincenzo Denaro

**Affiliations:** ^1^ Research Unit of Orthopaedic and Trauma Surgery Fondazione Policlinico Universitario Campus Bio‐Medico Roma Italy; ^2^ Research Unit of Orthopaedic and Trauma Surgery, Department of Medicine and Surgery Università Campus Bio‐ Medico di Roma Roma Italy; ^3^ Department of Medical and Surgical Sciences University of Catanzaro “Magna Grecia” Catanzaro Italy; ^4^ Department of Orthopaedic Surgery and Sportsmedicine, Aspetar Hospital Orthopaedic Surgeon and Assistant Chief of Surgery for Research Doha Qatar; ^5^ CNR‐IASI, Laboratorio di Biomatematica, Consiglio Nazionale delle Ricerche Istituto di Analisi dei Sistemi ed Informatica Rome Italy; ^6^ Department of Orthopaedics, Institute of Clinical Sciences, The Sahlgrenska Academy University of Gothenburg Gothenburg Sweden; ^7^ Clinica Ortopedica e Traumatologica II IRCCS Istituto Ortopedico Rizzoli Bologna Italy

**Keywords:** ankle, arthrodesis, arthroplasty, epidemiology, surgery

## Abstract

**Purpose:**

The goal of this study was to assess the annual incidence of tibiotarsal arthrodesis in Italy and the epidemiological characteristics of patients requiring surgery. A secondary aim was to assess the economic impact of this type of surgery on the healthcare system.

**Methods:**

The analysis was conducted by using the National Hospital Discharge Records (NHDR) database provided by the Italian Ministry of Health.

**Results:**

8380 tibiotarsal arthrodeses were performed in Italy. One procedure per 100,000 adult Italians was the cumulative incidence. The age group of 60–64 years required the highest number of procedures. Males represented the majority of patients undergoing surgery (59.6%). The median length of hospital stay was 7.3 ± 10.8 days. On average, older patients had more days of hospitalization. Main primary diagnosis codes according to the ICD‐9‐CM were: 715.27 (17.5%); 905.4 (15.8%); 715.17 (11.7%); 716.17 (5.1%); 733.82 (3.9%); 718.47 (3.4%). The admission reimbursement in Italy ranges from 1887€ to 4405€ depending on the length of stay. In 16 years, a total expenditure of 36,803,844€ was projected. The procedure in Italy costed an average of 2,300,240 ± 373,514€ per year.

**Conclusions:**

The socioeconomic impact of tibiotarsal arthrodesis in Italy is relevant. Between 2001 and 2016, the frequency of this procedure progressively rose. Patients undergoing surgery in Italy are mainly men, still active and part of the working population. Nationwide epidemiologic studies are useful to understand the present and future development of the management of ankle diseases.

**Level of Evidence:**

Level III.

**Abbreviations**
DRG: diagnosis‐related groups
ICD‐9‐CM: International Classification of Diseases, Ninth Revision, Clinical Modification
ISTAT: National Institute for Statistics
NHDR: national hospital discharge records
NHS: national health service
SDO: national hospital discharge records

## INTRODUCTION

Three articular surfaces constitute the ankle joint: [3] the weight‐bearing articulation of the talar dome and the distal tibial plafond, the lateral articulation between the medial fibular malleolus and lateral talar body, and the medial articulation between the lateral tibial malleolus and the medial body of the talus [3]. In the literature, treatment of ankle arthritis continues to be a hot topic of discussion. Ankle arthrodesis or total ankle replacement have traditionally been the preferred definitive surgical treatments [19]. Although ankle arthrodesis, open or arthroscopic, was once considered to be the ‘gold standard’, use of total ankle replacement has recently risen [22, 27]. Chronic ankle pain refractory to conservative treatment and restricting daily activities is the main indication for surgery [3]. However, successful patient selection and preparation are essential [5]. Ankle arthritis is frequently posttraumatic: even after well executed open/closed reduction and internal/external fixation of ankle fractures, many of these joints can gradually advance to late degenerative stage [3, 5]. Other less common indications are: severe pes planus for posterior tibial tendon dysfunction; talus avascular necrosis; joint destruction for rheumatoid arthritis; diabetic neuropathic osteoarthropathy; salvage in case of failed total ankle replacement surgery [3, 5]. A nationwide study in the State of New York from 2009 to 2018 showed an increase of 25% for ankle arthrodesis, whereas ankle arthroplasty increased by 757% [4]. Several studies have demonstrated that, when indicated, ankle fusion with internal fixation has a good success rate [6, 7, 20]. However, bleeding, soft tissue infection, osteomyelitis, short‐ or long‐term nerve damage, vascular injury, nonunion, malunion, and donor site morbidity are all examples of postoperative problems [8, 12]. In the last decades, total ankle replacement has grown in popularity as a treatment option for end‐stage ankle arthritis: although more expensive than ankle arthrodesis, total ankle replacement showed reduced revision and complication rates [27]. In the current literature, there is a lack of information about these data in the Italian population. One of the central principles of the Italian National Health Service (NHS) is equity in access to healthcare; patients in Italy are given free admission to the NHS. This study aimed to estimate the annual number of tibiotarsal arthrodesis in Italy and the patients' epidemiological aspects from 2001 to 2016 on the basis of official data sources, such as hospitalization records. A secondary aim was to assess the economic impact on the healthcare system of tibiotarsal arthrodesis. The analysis of this data may be vital to better address the future of this surgery and the related health service planning.

## MATERIALS AND METHODS

The analysis of present study is based on the National Hospital Discharge records (SDO), an official database provided by the Italian Ministry of Health containing data from all Italian private and public hospitals. Diagnoses are coded according to the International Classification of Diseases, Ninth Revision, Clinical Modification (ICD‐9‐CM).

These records comprise the patient's age, sex, domicile, the region of hospitalization, length of the hospitalization, diagnoses and procedures. Individual‐level research data are available from 2001 to 2016. The tibiotarsal arthrodesis procedure was defined by the 81.11 ICD‐9‐CM code. Data about population size were found from the National Institute for Statistics (ISTAT). The incidence rates were also stratified by year, age group and gender. All analyses refer to Italian adult population, that is, patients over 15 years of age.

Inclusion criteria: all patients included in the present study underwent a tibiotarsal arthrodesis procedure. Exclusion criteria: exclusion was applied when a diagnosis code associated with that of tibiotarsal arthrodesis procedure was atypical and did not apply to the 81.11 code according to the ICD‐9‐CM.

### Statistics

A series of descriptive statistical analyses was carried out by means of R program, a software environment for statistical computing and graphics. All statistical analyses were performed with IBM SPSS Statistics for Windows, Version 26.0. Armonk, NY: IBM Corp and Microsoft Excel (2019). Continuous and categorical data were summarized as means and standard deviations and frequencies and percentage as appropriate. The incidence rates were calculated dividing the number of tibiotarsal arthrodesis procedures by the annual adult population size (ISTAT data) per 100,000.

Analyses of estimated costs were based on the cost ascribed to diagnosis‐related groups (DRGs), according to Ministerial Decree (18 December 2008). In Italy, reimbursement is the same for all the procedures under DRGs, regardless of the diagnosis, the complexity of the procedure, or the patient's health status at admission. Economic reimbursement has been calculated per single year as an average between the minimum and maximum potential reimbursement value. In Italy, indeed, reimbursement varies from region to region, hence explaining these ranges of excursion.

## RESULTS

### Demographics

**Figure 1:**
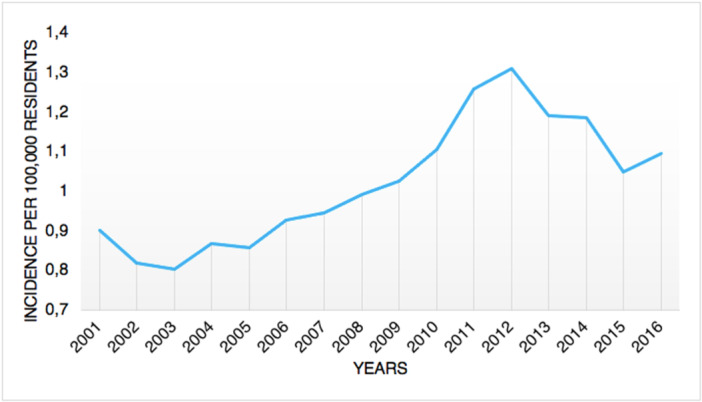
Incidence of tibiotarsal arthrodesis per 100,000 residents between 2001 and 2016.

**Figure 2:**
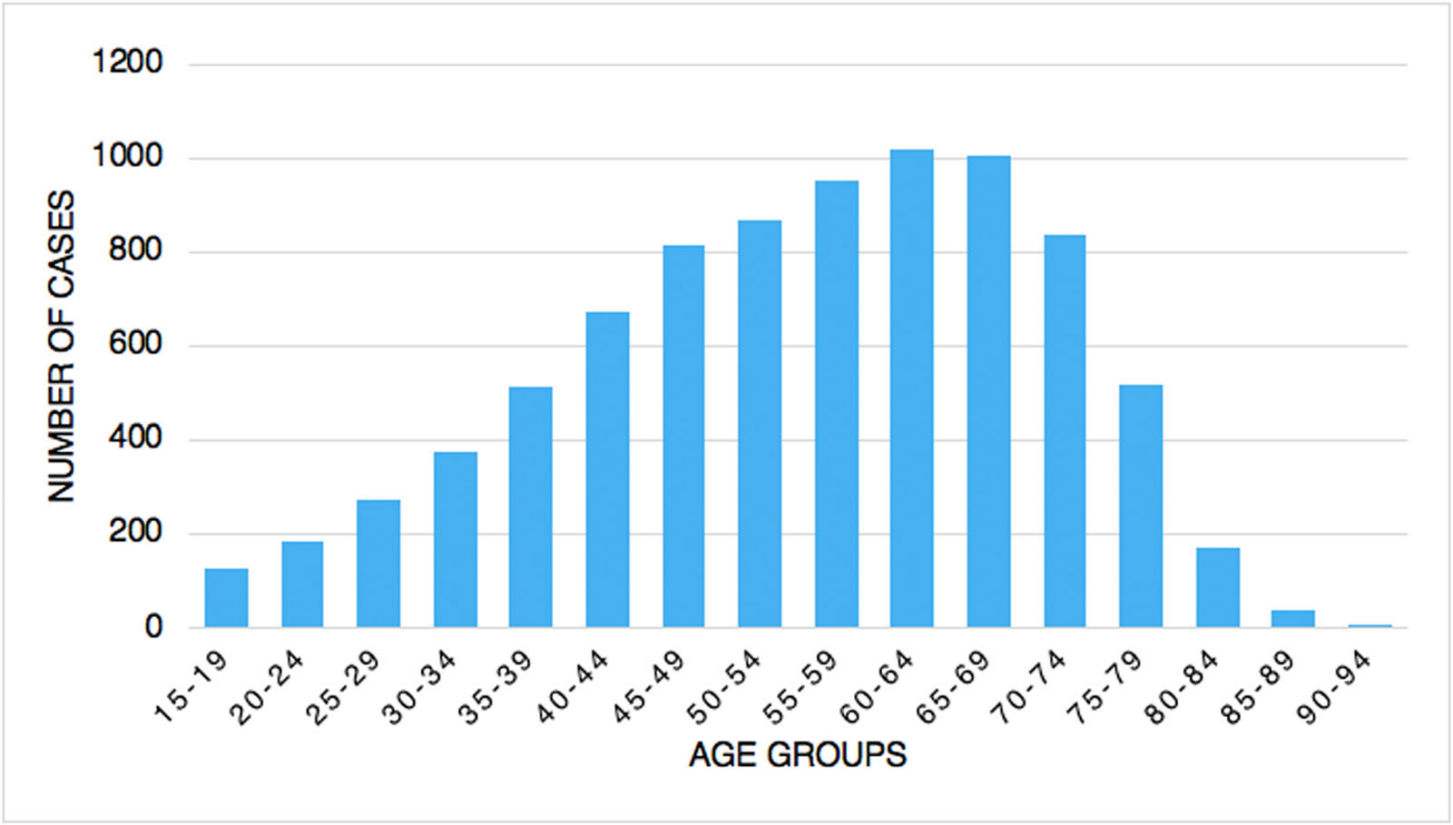
Number of tibiotarsal arthrodesis by age groups.

**Figure 3:**
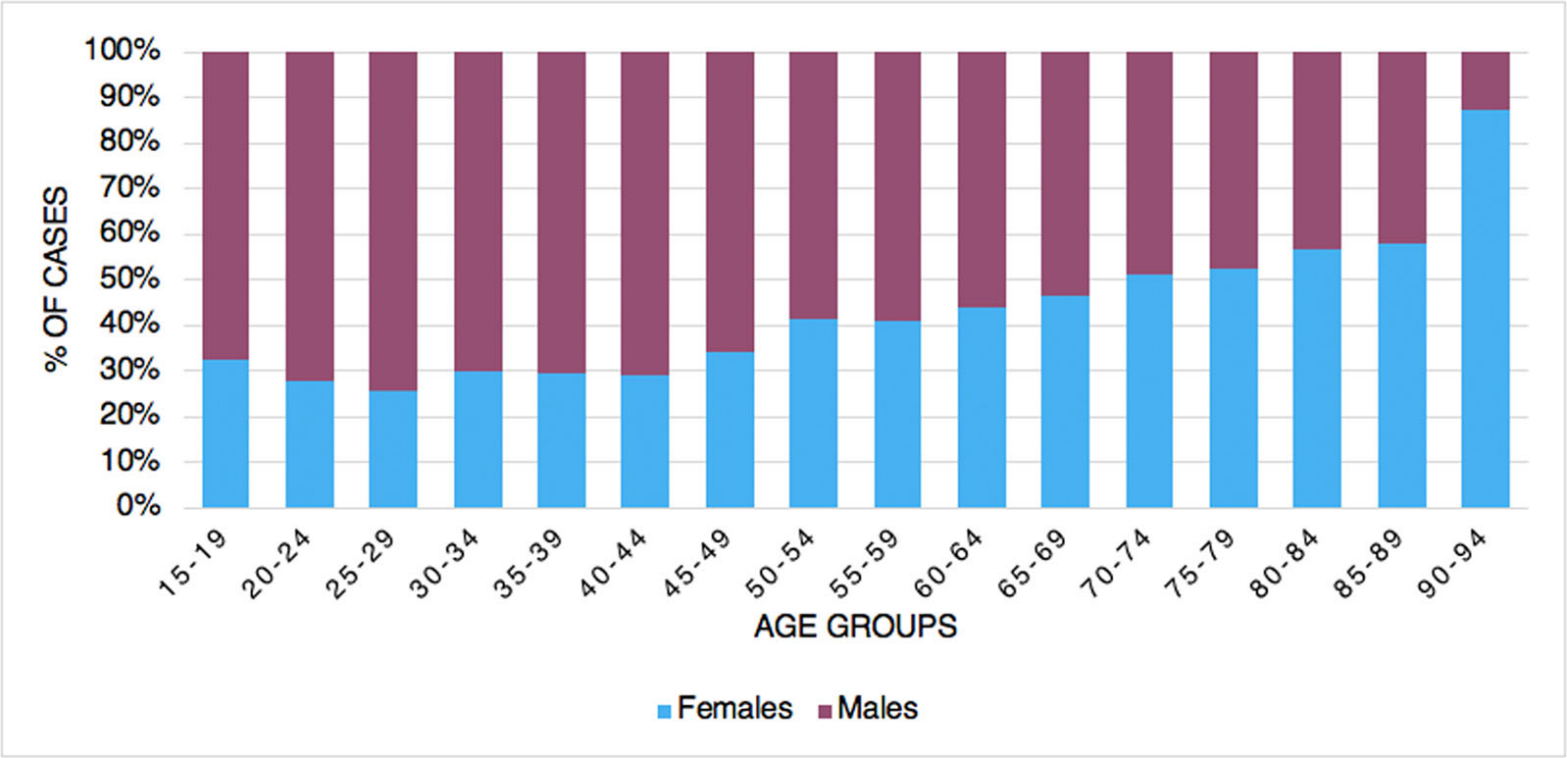
Percentages of tibiotarsal arthrodesis by age group and gender.

**Figure 4:**
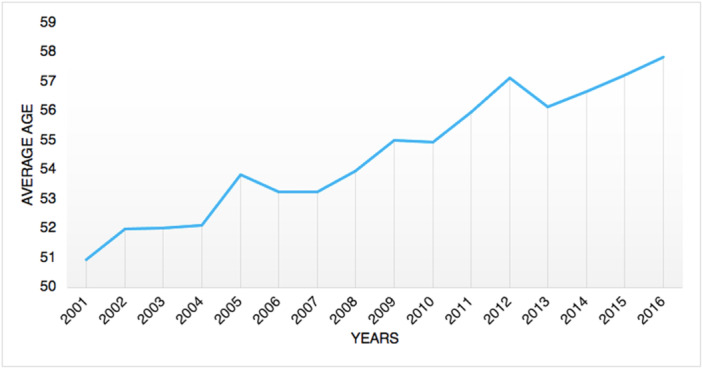
Average age over the years.

From 2001 to 2016, 8380 tibiotarsal arthrodeses were performed in Italy, with a peak of 674 procedures in 2012. The cumulative incidence was one procedure for every 100,000 adult Italian inhabitants. From 2001 to 2016, the incidence of operations increased from 0.9 to 1.1 per 100,000 residents (Figure 1). Overall, the highest number of procedures was found in the 60‐ to 64‐year age group (Figure 2). Males represented the majority of patients undergoing tibiotarsal arthrodesis (females 40.4% and males 59.6%). Between 70–74 and 90–94 age classes, a higher percentage of females was shown (Figure 3). From 2001 to 2016, the average age of patients was 54.8 ± 15.4 with an increasing trend (Figure 4).

### Length of the hospitalization

**Figure 5:**
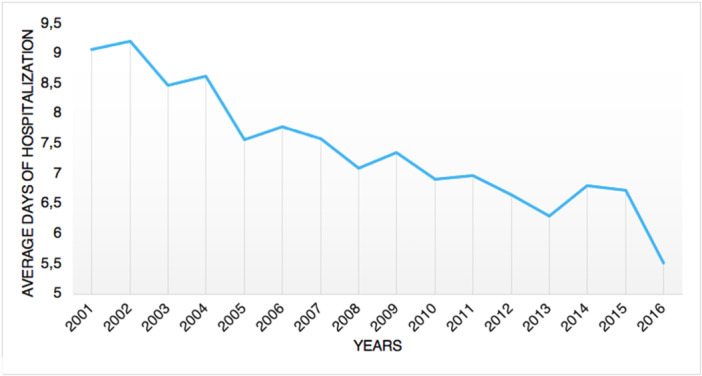
Average days of hospitalization over the years.

**Figure 6:**
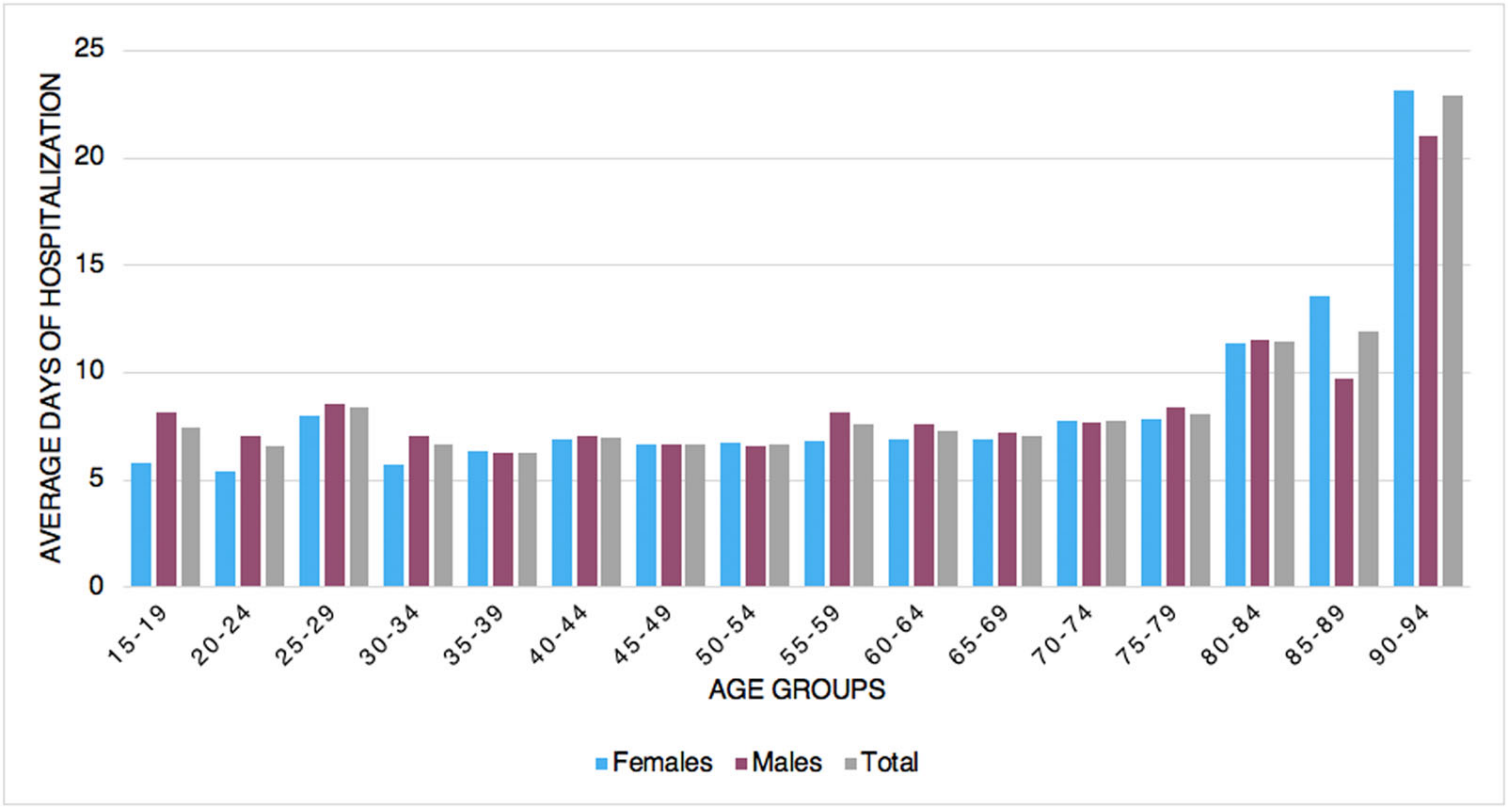
Average days of hospitalization by gender and age groups.

The median length of hospital stay was 7.3 ± 10.8 days, with a decreasing trend (Figure 5). Males had, on average, more days of hospitalization than females (females 7.2 ± 10.9 days and males 7.4 ± 10.8 days). On average, older patients had more days of hospitalization (Figure 6).

### Main primary diagnoses

During the 16‐year study period, the main primary diagnoses were: Osteoarthrosis, localized, secondary, ankle and foot (17.5%, ICD‐9‐CM code: 715.27); late effect of fracture of lower extremities (15.8%, ICD‐9‐CM code: 905.4); osteoarthrosis, localized, primary, ankle and foot (11.7%, ICD‐9‐CM code: 715.17); traumatic arthropathy, ankle and foot (5.1%, ICD‐9‐CM code: 716.17); nonunion of fracture (3.9%, ICD‐9‐CM code: 733.82) and contracture of joint, ankle and foot (3.4%, ICD‐9‐CM code: 718.47).

### Economic impact

**Figure 7:**
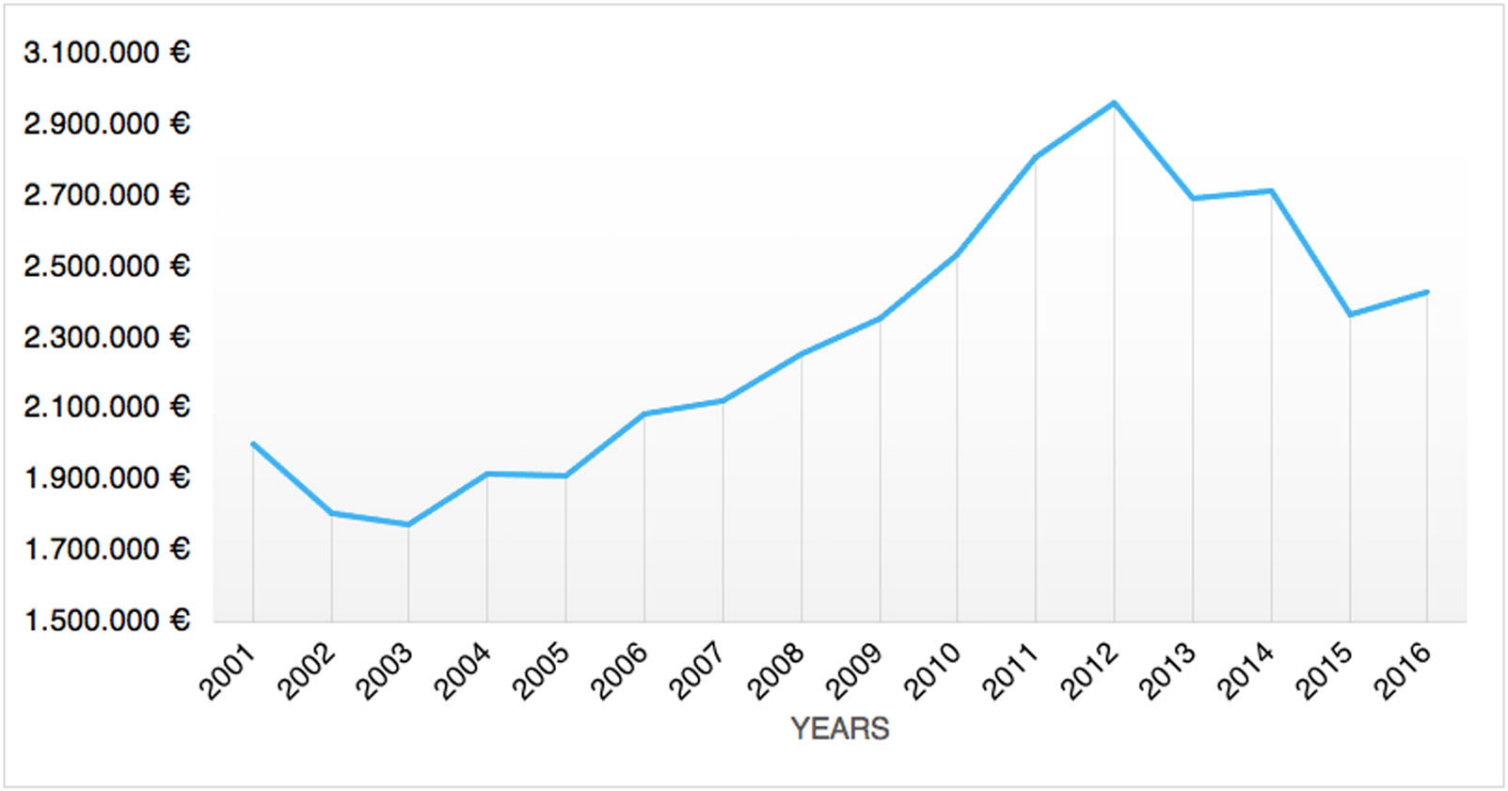
Annual burden of tibiotarsal arthrodesis procedures.

The average hospital reimbursement in Italy ranges from 4405€ (more than 1‐day stay, with a 20€ rise for each additional day of hospitalization) to 1887€ (1‐day stay treatment) for each tibiotarsal arthrodesis hospital admission. Between 2001 and 2016, a total expenditure of 36,803,844€ was projected. Tibiotarsal arthrodesis procedures in Italy cost an average of 2,300,240 ± 373,514€, with costs ranging from 1,776,456€ in 2003 to 2,967,192€ in 2012 (Figure 7).

## DISCUSSION

This study is the first to outline tibiotarsal arthrodesis surgical trends in Italy from 2001 to 2016. The epidemiology of patients requiring ankle surgery should be carefully considered when evaluating treatment options. Due in major part to recent improvements in surgical procedure and implant design, ankle arthroplasty has become a viable approach to treat ankle osteoarthritis [13, 14]. Increasing implant survivability, better biomechanical outcomes and equal pain relief have been shown by ankle arthroplasty when compared to ankle fusion [13, 14]. In a systematic review by Fanelli et al. [11], no differences in risk of complications, risk of re‐operations and postoperative functional outcomes between ankle arthroplasty and ankle fusion were observed. However, patients undergoing ankle arthroplasty showed better health‐related quality of life than ankle arthrodesis.

In the present study, the age group 60–64 had the largest prevalence of ankle fusion procedures, with a substantial influence on the working population. Tucker et al. recorded the largest prevalence of ankle fusion surgery in patients between 65 and 75 years of age [27]. Data from the State of New York registry showed a mean age of 58.1 for patients undergoing ankle arthrodesis [4]. Findings from the current study showed a lower average age of patients undergoing surgery (54.8 ± 15.4 with an increasing trend). A study on the Finnish Care Register for Health Care reported that from 1997 to 2018, a three‐fold increase in the incidence of ankle replacement was observed [26]. Conversely, the incidence of ankle fusion progressively decreased over the study period [26]. Putting together foot and ankle fusion, Best et al. [2] described an increase by 146% in incidence rate from 1994 to 2006 in the United States. The present study reported a different situation in Italy, with an increase in the need for ankle fusion from 2001 to 2012; from 2012 to 2016, a decreasing incidence pattern was observed.

Despite the management of end‐stage ankle arthritis remains a topic under debate in the orthopaedic community, the epidemiological analysis of the present study highlighted that Italian patients requiring surgery are relatively young and active. It remains unclear whether, in some of these patients, ankle arthroplasty might have provided better functional results than ankle fusion. Although open surgery is the conventional option for ankle fusion, there are some reports in the literature regarding the use of the arthroscopy procedure with encouraging results [18]. However, a recent epidemiological study reported a decreasing request for ankle arthroscopy among Italian surgeons [17].

The majority of patients undergoing tibiotarsal arthrodesis in Italy were males (59.6%). Interestingly, Brodeur et al. highlighted that patients undergoing ankle arthrodesis were more likely to be male, African American or lived in areas of social deprivation when compared to patients undergoing ankle arthroplasty [4]. Other registry analyses showed a higher prevalence of ankle arthritis diagnoses in females but equal requirement for ankle fusion surgery between genders [27].

Data from the State of New York registry revealed that, on average, ankle fusion surgery requires longer length of stay compared to ankle arthroplasty [4]. The present study showed a decreasing trend of the median length of hospital stay. Similarly, a study by Pugely et al. [22] in the United States showed that length of stay dramatically decreased from 8.7 days in 1991 to 2.3 days in 2010 in ankle replacement and from 5.5 to 3.2 days in ankle fusion. Those patterns could be explained by hospitals generally rearranging the length of stay in order to increase the healthcare efficiency. It might also be related to the advancement of minimally invasive surgical methods and the speedier recovery following anaesthetic treatments throughout time. No data, however, could support this assertion.

It is generally accepted that chronic ankle pain refractory to conservative treatment is the main indication for ankle fusion surgery [3]. In line with the literature [3, 5, 23], in this analysis, primary and secondary osteoarthritis of the ankle represented the leading causes requiring surgery. Fracture sequelae represented other important conditions treated with ankle arthrodesis.

The economic burden of ankle arthrodesis in Italy has been shown in the economic analysis of the present study. The admission reimbursement for tibiotarsal arthrodesis in Italy ranges from 1887€ to 4405€ depending on the length of stay. In the United States, it amounts to $6962.99, as evaluated by Tucker et al. [27]. An important finding of the current study is the growing rate of surgical procedures in Italian hospitals between 2001 and 2016, indicating an increase in the socioeconomic cost of tibiotarsal arthrodesis. It is confirmed by the average annual cost spent by the Italian healthcare system for tibiotarsal arthrodesis that has almost doubled from 2003 to 2012. Literature shows that ankle arthrodesis is a procedure generally less expensive than ankle arthroplasty [9, 27]. However, patients with ankle fusion experience well‐known significant functional deficits, including trouble walking on uneven surfaces and ankle pain from prolonged standing [21, 25]. For these reasons, it has been claimed that joint replacement in ankle arthritis with the proper criteria may be recognized as the standard of care [9]. Moreover, it is possible to successfully execute an ankle arthrodesis utilizing a variety of procedures when an ankle arthroplasty fails for whatever reason [1, 15]. In contrast, several studies highlighted that complication and revision rates of ankle replacement are generally higher than tibiotarsal arthrodesis [10, 16, 24]. Moreover, in terms of direct healthcare costs, ankle arthroplasty is a more expensive treatment option than ankle fusion, though indirect costs due to productivity loss and ankle disability are hard to estimate [9, 27].

This study has some limitations. First, this study's usage of the ICD‐9‐CM, which is the basis for all reported diagnoses and procedures, is based on administrative data from various hospitals and areas. Because of the numerous hospitals involved, it is hard to identify diagnoses or coding inaccuracies. Second, the absence of outcome scores may be a limitation of the current study: because hospitalizations are anonymous in the Italian healthcare system, patients do not acquire a specific ID number. In other words, patients who underwent multiple surgical procedures could have been counted twice or more. Third, there might be inter‐observer discrepancies because the ICD‐9 classification was done by surgeons. Fourth, a potential limitation of the economic analysis is given by the wide variability of reimbursements from region to region in Italy for the same procedure. Accordingly, it is not possible with our data to determine the exact costs afforded by the National Health Care System. In order to overcome this limitation, we have estimated an average value between the minimum and the maximum cost. Fifth, the operative treatment code 81.11 was considered in the present study. It stands for ankle arthrodesis, however, it can be used for both open and arthroscopic knee procedures. Therefore, the generic fashion of the ICD9‐CM resulted in the impossibility to differentiate between open and arthroscopic approaches used for ankle arthrodesis performed during the study period.

## CONCLUSIONS

In conclusion, ankle replacement and tibiotarsal arthrodesis are valid treatment options for many end‐stage ankle conditions. Both these procedures have their pros and cons in terms of costs, short‐term and long‐term functional results. Thus, management of severe ankle arthritis is still under debate. This study sought to determine the epidemiology of patients requiring tibiotarsal arthrodesis in Italy using official data sources including hospitalization records. A secondary aim was to assess the economic impact on the healthcare system of tibiotarsal arthrodesis. Results of the current study showed the increasing trend of tibiotarsal arthrodesis procedures in the management of ankle disease in Italy. Nonetheless, in the literature mentioned, it was noted that there is still no known international consensus regarding the specific indications between ankle arthroplasty and tibiotarsal arthrodesis. Nationwide epidemiologic studies are useful to understand the actual burden of this type of surgery on the healthcare system and its future development in the management of ankle diseases.

## AUTHOR CONTRIBUTIONS

**Umile Giuseppe Longo**: Conceptualization; methodology; formal analysis; investigation; data curation; writing—review and editing; supervision; project administration. **Rocco Papalia**: Conceptualization. **Alessandro Mazzola**: Methodology; formal analysis; writing—original draft preparation. **Alessandra Corradini**: Methodology; writing—original draft preparation. **Alessandro De Sire**: Methodology; investigation; visualization; supervision. **Pieter D'Hooghe**: Formal analysis; investigation; visualization. **Ilaria Piergentili**: Software validation; formal analysis. **Kristian Samuelsson**: Software validation; formal analysis; writing—review and editing. **Stefano Zaffagnini**: Data curation. **Vincenzo Denaro**: Writing—original draft preparation; writing—review and editing; project administration. All authors have read and agreed to the published version of the manuscript.

## CONFLICT OF INTEREST STATEMENT

Kristian Samuelsson is a member of the Board of Directors of Getinge AB (publ) and medtech advisor to Carl Bennet AB. The other authors declare no conflicts of interest.

## ETHICS STATEMENT

The Institutional Review Board of Campus Bio‐Medico University of Rome ruled that no formal ethics approval was required in this particular case and the need to obtain informed consent was waived based on the retrospective design and anonymization of patient identifiers (Prot. number: 113/20 [OSS] ComEt UCBM). All methods were performed in accordance with the relevant guidelines and regulations. All data were obtained by the Direzione Generale della Programmazione Sanitaria—Banca Dati SDO of the Italian Ministry of Health.

## Data Availability

The data sets used and/or analysed during the current study are available from the corresponding author on reasonable request. The access to the database is on request. All data were obtained by the Direzione Generale della Programmazione Sanitaria—Banca Dati SDO of the Italian Ministry of Health.
